# The use of single-stapling techniques reduces anastomotic complications in minimal-invasive rectal surgery

**DOI:** 10.1007/s00384-022-04197-5

**Published:** 2022-06-15

**Authors:** Maximilian Brunner, Alaa Zu’bi, Klaus Weber, Axel Denz, Melanie Langheinrich, Stephan Kersting, Georg F. Weber, Robert Grützmann, Christian Krautz

**Affiliations:** 1 Department of General and Visceral Surgery, Friedrich-Alexander-University, Krankenhausstraße 12, Erlangen, 91054 Germany; 2 Department of General, Thoracic and Vascular Surgery, Greifswald University, Ferdinand-Sauerbruch-Straße, VisceralGreifswald, Germany

**Keywords:** Rectal anastomosis, Single-stapling technique, Double-stapling technique, Colorectal cancer, Colorectal resections, Crossing stapler lines

## Abstract

**Background:**

Leakage of rectal anastomoses is one of the most important and feared complications in colorectal surgery. Apart from patient-specific risk factors, technical aspects may influence the occurrence of anastomotic complications. This study investigated whether using single-stapling techniques (SST) instead of the double-stapling technique (DST) for minimal-invasive rectal anastomosis is associated with a lower rate of anastomotic complications.

**Methods:**

A retrospective review of 272 patients who received a minimally invasive stapled rectal anastomosis (3–16 cm from the anal verge) at our institution from 2015 to 2020 was performed. In 131 patients, rectal anastomosis was created by SST (SST group), while 141 patients received a rectal anastomosis with crossing stapler lines (DST group). The impact of the anastomotic technique on patient outcomes was determined by uni- and multivariate analyses.

**Results:**

Overall anastomotic leakage rate was 6%. Patients with SST anastomoses had a lower leakage rate than patients with DST anastomoses (3% vs. 9% in the DST group, *p* = 0.045). The rate of anastomotic stenosis was lower in the SST group than in the DST group (1% vs. 6%, *p* = 0.037). Overall morbidity and mortality did not differ between the two groups. Multivariate analysis showed that single-stapling techniques significantly reduce the risk of anastomotic leakage (OR 3.5 [1.0–11.5], *p* = 0.043).

**Conclusion:**

The use of SST for rectal anastomosis may help to reduce anastomotic complications. This finding should be confirmed by a randomized controlled trial.

**Supplementary Information:**

The online version contains supplementary material available at 10.1007/s00384-022-04197-5.

## Introduction

Due to the ongoing technical progress and a growing body of evidence on the advantages of minimal-invasive approaches (lower intraoperative blood loss, shorter hospital stay, lower postoperative pain medication requirement, lower incisional hernia risk, comparable oncological outcome), the number of minimal-invasive colorectal resections is steadily increasing [1, 2].

One of the most critical complications following colorectal resections is the occurrence of anastomotic leakage due to relevant clinical and economic consequences. Affected patients may face severe sequelae such as reoperation, prolonged hospital stay, higher risk of accompanying morbidities, and inability to undergo necessary adjuvant chemotherapy [3, 4]. In addition, a meta-analysis from 2011 including a total of 12,202 patients with rectal cancer showed that anastomotic leakage is associated with a significantly higher local recurrence rate (hazard ratio 2.05 [95% Cl: 1.5–2.8], *p* = 0.0001) [5].

According to the literature, rates of anastomotic leakage reported for colorectal resections vary between 3 and 20% [3]. In addition to patient-specific risk factors (such as the presence of malnutrition, nicotine and alcohol abuse, steroid use, leukocytosis, previous cardiovascular diseases as well as a high ASA score) surgical aspects play a decisive role in the development of anastomotic leakage [6–8]. Currently, the “classical” double-stapling technique (DST) is the most widely used method for minimal-invasive colorectal anastomoses [9–11]. For double-stapling anastomosis, the rectal stump is transected using a linear stapler. After that, a circular stapler is used to create the anastomosis between the descending colon and the transected rectum [12, 13]. This technique results in intersecting stapler lines and the formation of “dog ears,” both considered predilection sites for leakages [14–16]. To increase the safety of minimal-invasive colorectal anastomoses and to reduce leakage rates, single-stapling techniques (SST) have been developed [17]. SST avoid intersections of stapler lines and the formation of “dog ears” but are more challenging to perform.

Until now, there are only a few studies that compared DST and SST in the setting of minimal-invasive colorectal surgery [18, 19]. Recently, two studies with limited patient cohorts (*n* = 100–120) showed ambiguous results (SST: 6% vs. DST: 8%; *p* = 0.695 [17] and SST: 10% vs. DST: 8%; *p* = 0.711 [19], respectively). Thus, we aimed to compare the rates of anastomotic complications (e.g., anastomotic leakage and stenosis) between SST and DST anastomoses in another patient cohort.

## Materials and methods

We retrospectively analyzed 272 consecutive patients who received a rectal anastomosis (3–16 cm from the anal verge) in minimal-invasive colorectal resections from 2015 to 2020 at the University Hospital Erlangen. Patients with rectal anastomosis below 3 cm from the anal verge were excluded, as in these patients the rectal stump is usually too short for single stapling techniques. Indications for surgery included malignancies as well as benign diseases like Crohn’s disease, diverticulitis, or endometriosis.

Data on patient demographics, comorbidities, neoadjuvant treatment, preoperative parameters, and intraoperative findings as well as on the postoperative course including morbidity were obtained and analyzed. Primary outcome was the occurrence of anastomotic leakage. Secondary endpoints included the occurrence of anastomotic stenosis, postoperative morbidity (according to Clavien-Dindo classification) and mortality, length of hospital stay, and readmission rate.

### Surgical techniques

All rectal anastomoses were performed based on three fundamental key points: (1) sufficient mobilization of the splenic flexure and rectal stump to obtain a tension-free anastomosis; (2) preservation of adequate blood perfusion of the descending colon and the rectal stump; (3) low-bleeding preparation through subtle hemostasis.

In patients with DST anastomosis, the rectal stump was closed using a linear stapler (45 or 60 mm) and the rectal anastomosis was created using a circular stapler (28 or 31 mm) [15, 16]. This technique resulted in unilateral or bilateral crossing stapler lines.

**Fig. 1:**
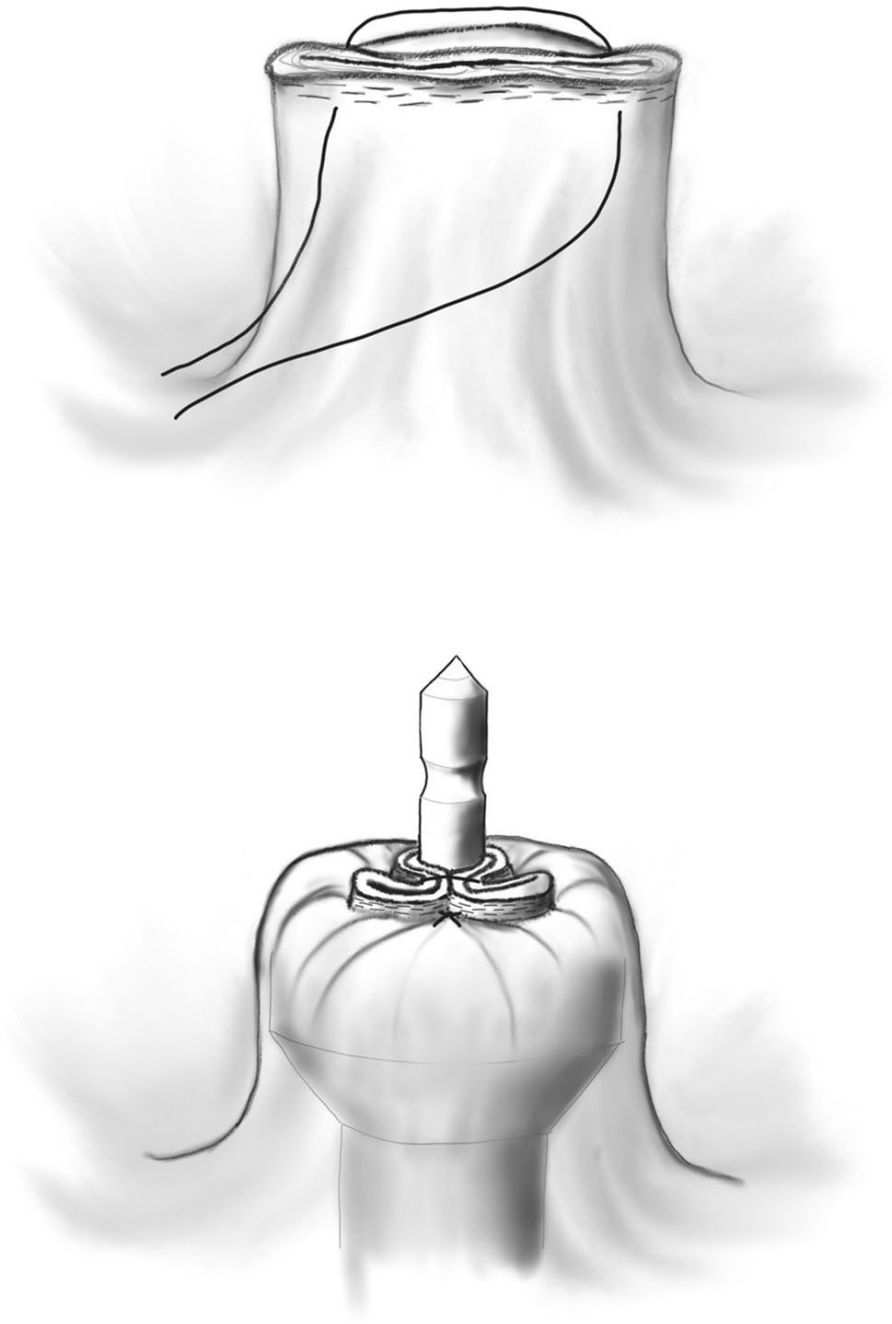
Omega suture: **a** thread placement and penetration site of the anvil rod (dot). **b** Ligation of the suture results in an omega shape of the linear stapler line, which is now located inside the round knife of the circular stapler

In patients with SST anastomosis, two different methods were used:

1. Omega suture:
  This technique was performed as previously described by Asao et al. [17]. After the rectum was divided with a linear stapler (45 or 60 mm), the circular stapler (28 or 31 mm) was placed allowing the anvil rod to penetrate the rectal stump near the linear stapler line. An omega suture including both ends of the linear stapler line was placed. The linear stapler line was approximated around the anvil rod of the circular stapler in an omega shape fashion as the omega suture was tied. This technique resulted in a complete resection of linear stapler line by the circular stapler (Fig. 1 and Fig. 2).

**Fig. 2:**
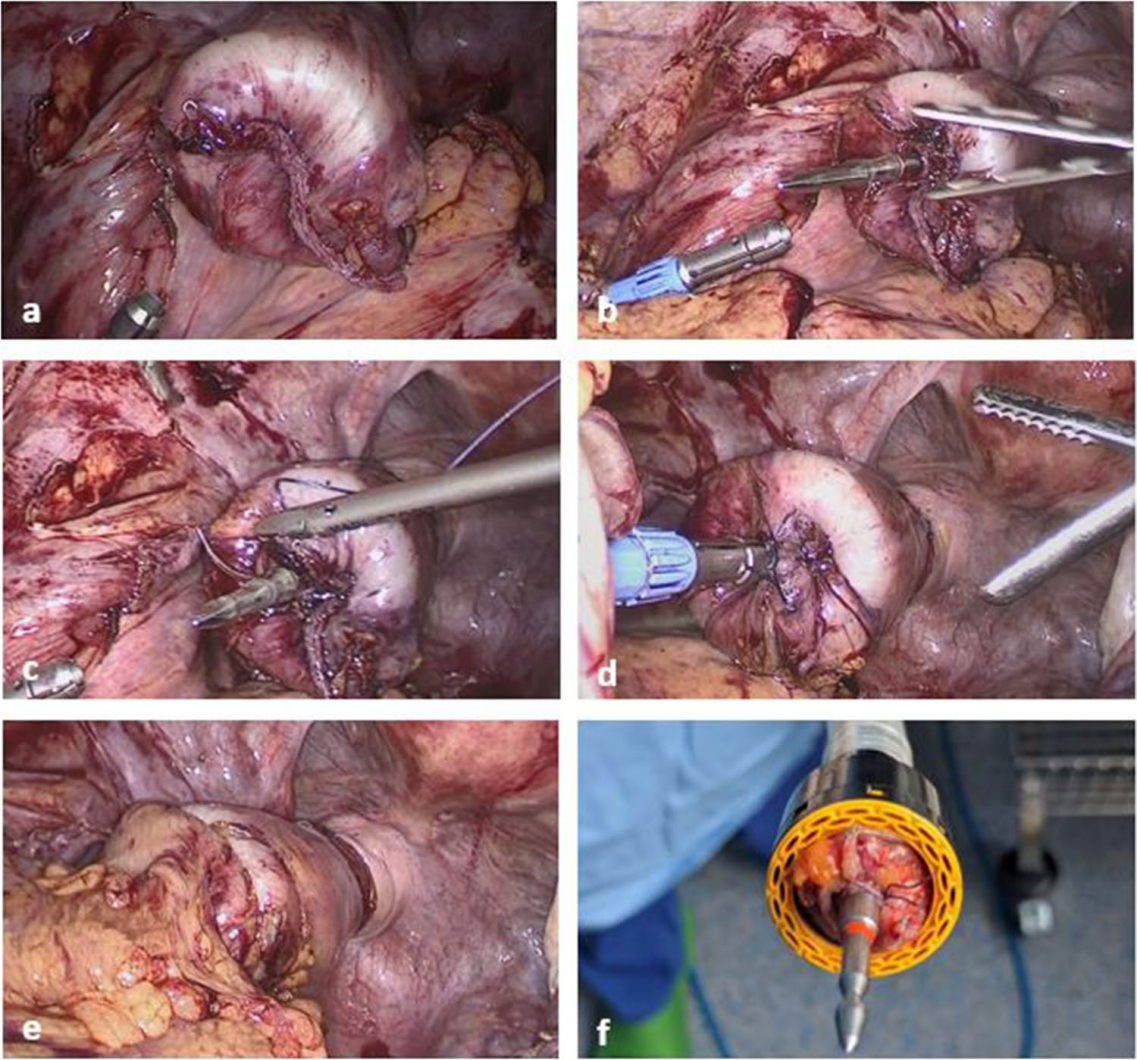
Single-stapling technique using the omega suture [16]: **a** after the rectum is divided with a linear stapler and a circular stapler is introduced through the anus. **b** The anvil rod penetrates the closed rectum at the median site of the linear stapler line. **c** A suture including both ends of the linear stapler line is placed. The thread is moved to the site of the anvil rod. **d** When the suture is tied, the linear stapler line is approximated around the anvil rod in an omega shape fashion. **e** The circular stapler is closed and fired, resulting in a complete resection of linear stapler line by the circular stapler. **f** Inspection of the distal stapler donut to verify complete resection of the linear stapler line (the suture and both ends of the linear stapler line are visible)
2. Purse-string suture:
  A laparoscopic reusable purse-string instrument and a polypropylene thread of size 2–0 were placed. The rectum was proximally ligated and cut just above the purse-string instrument. Alternatively, a handsewn purse-string suture with a 2–0 polypropylene thread was performed. After the introduction of the circular stapler, the purse-string suture was closed around the anvil rod. The anvil rod and head were connected, and the anastomosis was performed using a circular stapler (28 or 31 mm).
  Regardless of the stapling technique, the stapler donuts were always inspected for completeness. Moreover, anastomotic integrity was always proofed by an intraoperative rectoscopy and an air leak test (transanal air insufflation with the pelvis filled with saline). A protective diverting ileostomy was performed for all rectal anastomosis in the lower third of the rectum and according to the surgeon’s preference. In all patients, a drain was inserted into the pelvis.

### Definition of anastomotic leakage

Postoperative anastomotic leakage of the rectal anastomosis was defined as the presence of one or both of the following criteria: (1) evidence of anastomotic leakage by rectoscopy or endoscopy; (2) radiological evidence of leakage by contrast-enhanced computer tomography.

### Definition of anastomotic stenosis

Postoperative anastomotic stenosis was defined as stricture of the rectal anastomosis requiring dilatation.

### Statistical analysis

Data analysis was performed with SPSS software (SPSS, version 24.0). Comparisons of metric and ordinal data were calculated with the Student’s *t*-test or Mann Whitney *U* test. The chi-square test was used for categorical data. Statistical significance was set at *p* < 0.05. Possible risk factors for an anastomotic leakage were determined by uni- and multivariate analysis.

## Results

### Demographics

Of the 272 patients (mean age: 54.1 years, 58% female) with a rectal anastomosis, SST was applied in 131 patients (SST group) and DST in 141 patients (DST group). Patients of the SST group were significantly older (56.9 vs. 51.6 years, *p* = 0.008) and suffered more often from arterial hypertension (40 vs. 28%, *p* = 0.040). All other demographic parameters did not significantly differ between the groups (Table 1).

**Table 1:** Patient demographics

	**All patients** ***n*** **= 272**	**SST** ***n*** **= 131**	**DST** ***n*** **= 141**	***p*-value**
Mean age (years) [range]	54.1 [23–86]	56.9 [23–85]	51.6 [23–86]	**0.008**
Sex				
Male	114 (42)	53 (41)	61 (43)	0.712
Female	158 (58)	78 (59)	80 (57)	
BMI (kg/m^2^) [range]	26.1 [16.7–49.9]	26.3 [17.7–49.9]	25.9 [16.7–38.5]	0.927
ASA				
1	44 (16)	14 (11)	30 (21)	0.062
2	196 (72)	100 (76)	96 (68)	
3	31 (11)	16 (12)	15 (11)	
4	1 (0)	1 (1)	0 (0)	
Active smoking	50 (19)	26 (20)	24 (17)	0.535
Immunosuppression/steroid therapy	11 (4)	7 (5)	4 (3)	0.364
Comorbidities				
Diabetes mellitus	25 (9)	11 (8)	14 (10)	0.681
Coronary heart disease	18 (7)	8 (6)	10 (7)	0.811
Heart insufficiency	10 (4)	4 (3)	6 (4)	0.751
Arterial hypertension	91 (34)	52 (40)	39 (28)	**0.040**
Preoperative radio- or/and chemotherapy	39 (14)	16 (12)	23 (16)	0.388
Previous abdominal surgeries [range]	1 [0–6]	1 [0–5]	1 [0–6]	0.655
Preoperative laboratory [range]				
White blood cell count (× 10^9^/l)	7.0 [2.0–15.7]	7.0 [2.0–15.0]	7.0 [3.0–15.7]	0.684
Hemoglobin (g/dl)	13.8 [8.0–17.4]	13.8 [8.0–17.4]	13.9 [9.7–16.9]	0.566
CRP (mg/l)	10 [0–186]	9 [0–147]	11 [0–186]	0.414
Creatinine (mg/dl)	0.8 [0.4–3.9]	0.8 [0.5–3.9]	0.8 [0.4–1.4]	0.530
Albumin (g/l) (*n* = 93)*	41.7 [26.0–51.5]	42.1 [35.5–48.8]	41.4 [26.0–51.5]	0.782

^*^Not always determined

### Surgical parameters

Colorectal malignancies (43%) followed by diverticulitis (32%) and endometriosis (21%) were the most common indication that necessitated the performance of a rectal anastomosis. Surgical procedures included rectal resections (51%), combined rectal and sigmoid resections (16%), sigmoid resections (29%), and left hemicolectomies (4%). Sixty-eight percent of all surgeries were performed laparoscopically and 32% robotically (Table 2). The SST was performed using the omega suture (70%) or the purse-string suture method (30%).

**Table 2:** Surgical parameters

	**All patients** **(*n*** **=** **272)**	**SST** **(*n*** **=** **131)**	**DST** **(*n*** **= 141)**	***p*-value**
Indication for surgery				
Oncological	116 (43)	59 (45)	57 (40)	**< 0.001**
Rectum	82 (71)	37 (63)	45 (79)	
Sigmoid	31 (27)	20 (34)	11 (19)	
Descending colon	3 (2)	2 (3)	1 (2)	
Non-oncological	156 (57)	72 (55)	84 (60)	
Diverticulitis	86 (55)	53 (74)	33 (39)	
Endometriosis	56 (36)	12 (17)	44 (52)	
Rectal/sigmoid adenoma	8 (5)	4 (6)	4 (5)	
Crohn´s disease	5 (3)	2 (3)	3 (4)	
Sigmoid perforation	1 (1)	1 (1)	0 (0)	
Operative time (min.)	297 [119–688]	295 [129–581]	300 [119–688]	0.822
Surgical approach				
Laparoscopic	184 (68)	85 (65)	99 (70)	0.366
Robotic	88 (32)	46 (35)	42 (30)	
Kind of surgery				
Rectal resection	139 (51)	55 (42)	84 (60)	**0.026**
Rectal + sigmoid resection	43 (16)	24 (18)	19 (14)	
Sigmoid resection	80 (29)	45 (34)	35 (25)	
Left hemicolectomy	10 (4)	7 (5)	3 (2)	
Ostomy	72 (27)	24 (18)	48 (34)	**0.004**
ICG	14 (5)	8 (6)	6 (4)	0.588
Level of anastomosis (cm) [range]	9 [3−16]	9 [3−16]	8 [3−16]	**0.004**
Intraoperative blood loss (ml) [range]	157 [10–1400]	132 [10–1000]	181 [10–1400]	**0.004**
Intraoperative intravenous fluids (ml) [range]	3359 [1000–10500]	3549 [1000–10500]	3187 [1000–7000]	0.060

Significant differences between the SST and the DST group regarding surgical parameters included more diverticulitis patients (40 vs. 23%, *p* < 0.001), less endometriosis patients (9 vs. 31%, *p* < 0.001), less rectal resections (42 vs. 60%, *p* = 0.026), a lower ostomy rate (18 vs. 34%, *p* = 0.004), a lower level of anastomosis (9 vs. 8 cm, *p* = 0.004), and less intraoperative blood loss (132 vs. 181 ml, *p* = 0.004) in the SST compared to the DST group (Table 2).

### Anastomotic leakage

In our cohort, 17 patients (6%) developed leakage of rectal anastomosis. Anastomotic leakage occurred significantly less often in the SST group compared to the DST group (4 patients (3%) vs. 13 patients (9%), *p* = 0.045).

### Primary endpoint analysis

In the univariate analysis, we identified three significant risk factors for postoperative anastomotic leakage (increase of operative time, *p* = 0.005; increase of intraoperative intravenous fluids, *p* = 0.031; use of DST, *p* = 0.045). Among these variables, the increase of operative time (OR 1.005 (95% CI = 1.000 – 1.011), *p* = 0.046) and the use of DST (OR 3.451 (95% CI = 1.039 – 11.462), *p* = 0.043) were confirmed as independent risk factors for the development of anastomotic leakage in the multivariate analysis (Table 3).

**Table 3:** Primary endpoint analysis (risk factors for anastomotic leakage)

**Variables**	**Univariate**			Multivariate*		
	**OR**	**95% CI**	***p*-value**	**OR**	**95% CI**	***p*-value**
Age (years)	1.011	0.979–1.043	0.515	-	-	-
Sex				-	-	-
Male	1.000					
Female	0.482	0.178–1.307	0.152			
BMI (kg/m^2^)	1.022	0.926–1.127	0.672	-	-	-
ASA				-	-	-
1/2	1.000					
3/4	1.000	0.218–4.590	1.000			
Smoking				-	-	-
No	1.000					
Yes	0.572	0.127–2.586	0.468			
Immunosuppression				-	-	-
No	1.000					
Yes	3.644	0.722–18.387	0.117			
Comorbidities**				-	-	-
No	1.000					
Yes	2.062	0.769–5.531	0.150			
Preoperative radio- or/and chemotherapy				-	-	-
No	1.000					
Yes	2.708	0.898–8.169	0.077			
Preoperative white blood cell count (× 10^9^/l)	1.106	0.907–1.349	0.319	-	-	-
Preoperative albumin (g/l)***	1.171	0.901–1.522	0.237	-	-	-
Indication for surgery				-	-	-
Oncological	1.000					
Non-oncological	0.498	0.184–1.350	0.171			
Operative time (Min.)	**1.007**	**1.002–1.012**	**0.005**	**1.005**	**1.000–1.011**	**0.046**
Protective ostomy				-	-	-
No	1.000					
Yes	1.562	0.556–4.390	0.398			
Level of anastomosis (cm)	0.932	0.816–1.063	0.292	-	-	-
Intraoperative blood loss (ml)	**1.001**	**0.999–1.003**	**0.263**	-	-	-
Intraoperative intravenous fluids (ml)	1.000	1.000–1.001	0.031	1.000	1.000–1.001	0.104
Stapling technique (SST vs. DST)						
SST	**1.000**					
DST	**3.225**	**1.024–10.156**	**0.045**	**3.451**	**1.039–11.462**	**0.043**

^*^Inclusion of parameters, if *p*-value was < 0.05 in univariate analysis; **including diabetes mellitus, coronary heart disease, heart insufficiency, and arterial hypertension; ***reduced number (*n* = 93) in analysis due to missing data

### Secondary endpoint analysis

In-hospital and 30-day morbidity and mortality as well as the rate of re-surgery did not differ between SST and DST groups. SST was associated with a significantly shorter length of hospital stay and a lower rate of readmission at 30 days compared to the DST group (8.7 vs. 9.4 days, *p* = 0.006 and 1% vs. 6%, *p* = 0.037, respectively). Compared to the DST group, anastomotic stenosis occurred significantly less often in the SST group (1 vs. 6%, *p* = 0.037) (Table 4).

**Table 4:** Secondary endpoint analysis (perioperative outcomes)

**Parameter**	**All patients (*n*** **= 272)**	**SST (*n*** **= 131)**	**DST (*n*** **= 141)**	***p*-value**
Morbidity (30 days)*				
I	8 (3)	1 (1)	7 (5)	0.120
II	21 (8)	11 (8)	10 (7)	
IIIa	3 (1)	1 (1)	2 (1)	
IIIb	18 (6)	5 (4)	13 (9)	
IV	3 (1)	2 (2)	1 (1)	
Overall	54 (20)	20 (15)	34 (24)	
Mortality (30 days)	1 (0)	0 (0)	1 (1)	1.000
Length of hospital stay	9.1 [5–51]	8.7 [5–51]	9.4 [5–39]	**0.006**
Readmission	9 (3)	1 (1)	8 (6)	**0.037**
Anastomotic leakage**	17 (6)	4 (3)	13 (9)	**0.045**
Anastomotic stenosis**	9 (3)	1 (1)	8 (6)	**0.037**

^*^According to Clavien-Dindo classification; **mean follow-up 36 months [range 4–75 months]

### Analysis of patients with postoperative anastomotic leakage

Patient demographics and outcomes in the patients with postoperative anastomotic leakage did not significantly differ between the stapling techniques (supplemental table 1). Eighty-two percent of all patients with anastomotic leakage that did not have a diverting ostomy required re-surgery. In contrast, patients with a diverting ostomy showed significant lower symptoms, a significant later diagnosis of anastomotic leakage as well as a significant lower rate of re-surgery (50 vs. 100%, *p* = 0.029; 8th vs. 44th POD, *p* = 0.005; 17 vs. 82%, *p* = 0.018).

## Discussion

The surgical technique plays a decisive role in avoiding anastomotic leakage following colorectal resections. Well-known surgical principles to prevent anastomotic leakage are gentle tissue handling, good hemostasis, adequate blood perfusion, asepsis and a tension-free anastomosis. Technical aspects that have been subject to critical debate in the literature are intersecting stapling lines and the formation of “dog ears” (everting corners of the rectal stump with potentially poor blood supply) in DST anastomoses. Both are considered weak spots that may play a role in the development of anastomotic leakage. SST has been introduced to overcome these structural disadvantages. An experimental investigation by Roumen et al. revealed that DST anastomoses have a lower bursting pressure than SST anastomoses. Notably, the “dog ears” were the first spots to burst under increasing intraluminal pressure [16]. In addition, low rates of anastomotic leakages have been reported for SST in open surgery [20].

The present study revealed a significantly lower rate of anastomotic leakage following minimal-invasive colorectal resections with SST anastomoses compared to those with DST anastomoses. Moreover, the multivariate analysis showed that DST is an independent risk factor for postoperative anastomotic leakage. In contrast, Kim et al. and Radonovic et al. found no significant differences among the leakage rates of patients with SST and DST anastomoses (SST: 6% vs. DST: 8%; *p* = 0.695 [18], SST: 10% vs. DST: 8%; *p* = 0.711 [19]). One possible explanation for these ambiguous results may lie in the method of how SST anastomoses are performed (e.g., using a purse-string suture vs. omega suture). While the studies mentioned above used purse-string sutures, we employed omega sutures in the majority of patients. The omega suture method has two advantages, which may have a favorable impact on anastomotic leakage rates. First, it prevents fecal spillage and bacterial contamination of the pelvic. Second, it is a simple single suture that is not as error-prone as a purse-string suture. In a sub-analysis, we did not find significant different leakage rates between purse-string suture SST and omega suture SST (data not shown). However, the number of patients in the purse-string suture group was low, which limits the validity of this finding.

There are some technical limitations for the use of the omega suture. First, the use of omega sutures can be challenging in ultra-low rectal anastomoses, especially in narrow pelvis of men. The robotic approach can help to overcome this limitation, but even with the robot the use of the omega suture is not always feasible. Second, long linear stapler line (more than two 45 mm or more than one 60 mm stapler firings) may not be completely located inside the round knife of the circular stapler (Fig. 1) resulting in an incomplete resection. Therefore, the use of an omega suture must be carefully considered, as a poorly performed omega sutures can facilitate anastomotic complications.

Additionally, our results confirmed that a longer operative time is associated with a higher rate of postoperative anastomotic leakages. This is in line with previous studies [21, 22]. However, other perioperative variables that have been reported in the literature, such as male gender, nicotine abuse, alcohol abuse, steroid use, leukocytosis, previous cardiovascular diseases, previous neoadjuvant treatment, the level of anastomosis as well as the ostomy rate, did not significantly affect leakage rates in our patient cohort [6–8, 22, 23].

Secondary endpoint analysis showed that the rate of anastomotic stenosis is significantly higher in DST than SST anastomoses. As anastomotic leakage is directly associated with the development of anastomotic stenosis [24], the latter may serve as a positive control. Therefore, the higher stenosis rate in the DST group may be regarded as indirect confirmation for the higher leakage rate in the DST group.

A sub-analysis of all patient cases with anastomotic leakage found no differences between the stapling techniques. Although this comparison is limited by the small number of patients, the data show that a diverting ostomy can mitigate the consequences of anastomotic leakage. These results underline the importance of a preoperative and intraoperative risk assessment regarding the development of anastomotic leakage.

The present study has several limitations. First, the retrospective design of our study may have incurred some bias. Second, the patient cohort is heterogeneous regarding the indications for surgery. Subsequently, the differences in leakage rates may be affected by the extent of resection and maintenance of the blood supply (e.g., the superior rectal arteria). In addition, the mean level of anastomosis was significantly lower in the DST group than in the SST group. However, the kind of surgery (oncological versus non-oncological) and the level of anastomosis were included in the risk assessment for anastomotic leakage and showed no significant association with the anastomotic leakage rate. Due to the limited number of patients in our study, homogenization of the groups using propensity score matching or subgroup analysis was not useful.

## Conclusion

Our results show that SST may significantly reduce rates of anastomotic leakage and stenosis. Randomized controlled trials are needed to confirm these findings.

## Supplementary Information

Below is the link to the electronic supplementary material.

Supplementary file1 (DOCX 23 KB)

## Declarations

### Statement on the welfare of animals

This article does not contain any studies with animals performed by any of the authors.
