# Supplementary material for: The use of single-stapling techniques reduces anastomotic complications in minimal-invasive rectal surgery
Source: Int J Colorectal Dis. 2022 Jun 15;37(7):1601–9. doi: 10.1007/s00384-022-04197-5 (PMC9262801; doi:10.1007/s00384-022-04197-5)
Supplement: Supplementary file 1 — Supplementary file1 (DOCX 23 KB) [file 384_2022_4197_MOESM1_ESM.docx]

**Supplemental Table:** Characteristics of patients with rectal anastomotic leakage

| **Parameter** | **SST**  **(n=4)** | **DST**  **(n=13)** | **p-value** |
| --- | --- | --- | --- |
| Kind of surgery  Rectal resection  Rectal + sigmoid resection  Sigmoid resection | 2 (50)  1 (25)  1 (25) | 9 (69)  1 (8)  3 (23) | 0.723 |
| Diverting ostomy | 0 (0) | 6 (46) | 0.237 |
| Level of anastomosis (cm) [range] | 9 [6-10] | 7 [3-16] | 0.412 |
| Diagnosis of anastomotic leakage  During hospital stay  On postoperative day | 3 (75)  9 [4-13] | 7 (54)  24 [3-103] | 0.603  0.956 |
| Due to symptoms | 4 (100) | 10 (77) | 0.541 |
| Treatment  Only antibiotics  Endoscopic irrigation +/- EndoVAC  Interventional drainage  Re-OP with lavage  Re-OP with diverting ostomy  Re-OP with Hartmann procedure | 0 (0)  0 (0)  0 (0)  0 (0)  3 (75)  1 (25) | 1 (8)  4 (31)  1 (8)  1 (8)  2 (15)  3 (23) | - |
| Need of re-surgery | 4 (100) | 6 (46) | 0.103 |
| Mortality | 0 (0) | 1 (8) | 1.000 |
| Presence of ostomy at last follow-up (n=15)* | 1 (33) | 2 (17) | 1.000 |

* Excluding two patients due to a cancer-related death (SST) and a complication-related death (DST)
